# Antiretroviral Therapy Enrollment Characteristics and Outcomes Among HIV-Infected Adolescents and Young Adults Compared with Older Adults — Seven African Countries, 2004–2013

**Published:** 2014-11-28

**Authors:** Andrew F. Auld, Simon G. Agolory, Ray W. Shiraishi, Fred Wabwire-Mangen, Gideon Kwesigabo, Modest Mulenga, Sebastian Hachizovu, Emeka Asadu, Moise Zanga Tuho, Virginie Ettiegne-Traore, Francisco Mbofana, Velephi Okello, Charles Azih, Julie A. Denison, Sharon Tsui, Olivier Koole, Harrison Kamiru, Harriet Nuwagaba-Biribonwoha, Charity Alfredo, Kebba Jobarteh, Solomon Odafe, Dennis Onotu, Kunomboa A. Ekra, Joseph S. Kouakou, Peter Ehrenkranz, George Bicego, Kwasi Torpey, Ya Diul Mukadi, Eric van Praag, Joris Menten, Timothy Mastro, Carol Dukes Hamilton, Mahesh Swaminathan, E. Kainne Dokubo, Andrew L. Baughman, Thomas Spira, Robert Colebunders, David Bangsberg, Richard Marlink, Aaron Zee, Jonathan Kaplan, Tedd V. Ellerbrock

**Affiliations:** 1 Division of Global HIV/AIDS, Center for Global Health, CDC; 2 Infectious Diseases Institute, Makerere University College of Health Sciences, Uganda; 3 Muhimbili University of Health and Allied Sciences, Tanzania; 4 Tropical Diseases Research Center, Zambia; 5 Ministry of Health, Nigeria; 6 Ministry of Health, Côte d’Ivoire; 7 National Institute of Health, Mozambique; 8 Ministry of Health, Swaziland; 9 Social and Behavioral Health Sciences, FHI 360, Washington, DC; 10 Institute of Tropical Medicine, Department of Clinical Sciences, Belgium; 11 International Center for AIDS Care and Treatment Programs-Columbia University, New York, NY; 12 Division of Global HIV/AIDS, Center for Global Health, CDC, Mozambique; 13 Division of Global HIV/AIDS, Center for Global Health, CDC, Nigeria; 14 Division of Global HIV/AIDS, Center for Global Health, CDC, Côte d’Ivoire; 15 Division of Global HIV/AIDS, Center for Global Health, CDC, Swaziland; 16 FHI 360, Zambia; 17 FHI 360, Haiti; 18 FHI 360, Tanzania; 19 Global Health, Population and Nutrition, FHI 360, Durham, NC; 20 Massachusetts General Hospital, Boston, MA; 21 Harvard School of Public Health, Boston, MA

Although scale-up of antiretroviral therapy (ART) since 2005 has contributed to declines of about 30% in the global annual number of human immunodeficiency (HIV)-related deaths and declines in global HIV incidence,* estimated annual HIV-related deaths among adolescents have increased by about 50% (1) and estimated adolescent HIV incidence has been relatively stable.† In 2012, an estimated 2,500 (40%) of all 6,300 daily new HIV infections occurred among persons aged 15–24 years.§ Difficulty enrolling adolescents and young adults in ART and high rates of loss to follow-up (LTFU) after ART initiation might be contributing to mortality and HIV incidence in this age group, but data are limited (2). To evaluate age-related ART retention challenges, data from retrospective cohort studies conducted in seven African countries among 16,421 patients, aged ≥15 years at enrollment, who initiated ART during 2004–2012 were analyzed. ART enrollment and outcome data were compared among three groups defined by age at enrollment: adolescents and young adults (aged 15–24 years), middle-aged adults (aged 25–49 years), and older adults (aged ≥50 years). Enrollees aged 15–24 years were predominantly female (81%–92%), commonly pregnant (3%–32% of females), unmarried (54%–73%), and, in four countries with employment data, unemployed (53%–86%). In comparison, older adults were more likely to be male (p<0.001), employed (p<0.001), and married, (p<0.05 in five countries). Compared with older adults, adolescents and young adults had higher LTFU rates in all seven countries, reaching statistical significance in three countries in crude and multivariable analyses. Evidence-based interventions to reduce LTFU for adolescent and young adult ART enrollees could help reduce mortality and HIV incidence in this age group.

In each of seven countries (Côte d’Ivoire, Nigeria, Swaziland, Mozambique, Zambia, Uganda, and Tanzania), a representative sample of ART facilities was selected using either probability-proportional-to-size sampling or purposeful (nonrandom) sampling (Table 1). At each selected facility, a sample frame of study-eligible ART patients was created, and simple random sampling used to select the desired sample size. Eligibility criteria included having started ART during 2004–2012 and ≥6 months before data abstraction. Data were abstracted from ART medical records onto standard forms.

Mortality and LTFU were the primary outcomes of interest. A patient was considered LTFU if he/she had not attended the facility in the 90 days preceding data abstraction for a medication refill, a laboratory visit, or a clinician visit. Mortality ascertainment occurred largely through passive reporting to the health facility by family or friends, and to a lesser extent, through country-specific tracing activities to locate patients late for clinic appointments.

Study design was controlled for during analysis. Age at ART initiation was divided into three age categories (3): 15–24 years, 25–49 years, and ≥50 years. Differences in demographic and clinical characteristics across age groups were assessed using chi-square tests for categorical variables and unadjusted linear regression models for continuous variables.

To estimate the association between age group and rates of death and LTFU, Cox proportional hazards regression models were used to estimate unadjusted and adjusted hazard ratios for each outcome separately. For the multivariable analysis, to best manage missing baseline demographic or clinical data, multiple imputation with chained equations was used to impute missing data included in the model (4). Twenty imputed datasets were created for each outcome: death and LTFU (4). The imputation model included the event indicator, all study variables, and the Nelson-Aalen estimate of cumulative hazard (4). The proportional hazards assumption was assessed using visual methods and the Grambsch and Therneu test.

Demographic and clinical characteristics of adults at ART initiation were compared across age groups by country (Table 2). Age distribution was relatively constant across countries, with 5%–16% aged 15–24 years, 70%–86% aged 25–49 years, and 8%–14% aged ≥50 years. In all seven countries, the youngest age group was almost exclusively female (81%–92%), and the middle-age group mostly female (60%–68%); in contrast, the oldest age group was mostly male in all countries, except Nigeria. In the six countries with data on pregnancy at ART enrollment, pregnancy prevalence was highest in the youngest age group in five countries, where it ranged from 16% to 32%. In all seven countries, being married or in a civil union was least common in the youngest age group (27%–46%), reaching statistical significance in five countries. In the four countries with data on employment status, the youngest age group was least likely to be employed at the time of ART enrollment (14%–47%) (p<0.05).

What is already known on this topic?

Although scale-up of antiretroviral therapy (ART) since 2005 has contributed to a decline of about 30% in the global annual number of human immunodeficiency (HIV)–related deaths and declines in global HIV incidence, estimated annual HIV-related deaths among adolescents have increased by about 50%, and estimated adolescent HIV incidence has been relatively stable. In 2012, an estimated 2,500 (40%) of all 6,300 daily new HIV infections occurred among persons aged 15–24 years. Difficulty enrolling adolescents and young adults in ART and high rates of loss to follow-up (LTFU) after ART initiation might be contributing to mortality and HIV incidence in this age group, but data are limited.

What is added by this report?

Age-related differences in enrollment characteristics and outcomes were analyzed among 16,421 patients aged ≥15 years starting ART in seven African countries (Côte d’Ivoire, Nigeria, Swaziland, Mozambique, Zambia, Uganda, and Tanzania) during 2004–2012. Patient characteristics and outcomes were compared across three age groups: adolescents and young adults (15–24 years), middle-aged adults (25–49 years), and older adults (≥50 years). Compared with older adults, adolescents and young adults had higher LTFU rates in all seven countries, reaching statistical significance in three countries (Côte d’Ivoire, Mozambique, and Tanzania) in both crude and multivariable analyses.

What are the implications for public health practice?

The higher risk for LTFU among adolescent and young adult ART enrollees, compared with older adults, increases their risk for death and increases the risk they will transmit HIV to seronegative sex partners. Effective interventions to reduce LTFU for adolescent and young adult ART enrollees could help reduce mortality and lower HIV incidence in this age group.

In all seven countries, median baseline weight was lowest in the youngest age group (48.2–58.0 kg), reaching statistical significance in six countries. In three countries (Nigeria, Swaziland, and Tanzania), prevalence of World Health Organization clinical stage 4 at ART initiation differed across age groups, tending to be lowest in the youngest and highest in the oldest age group (p<0.05). Median baseline CD4 count was similar across age groups in all countries, except Nigeria, where the median was highest in the youngest age group (p=0.004). Median baseline hemoglobin was significantly lower in the youngest age group in four countries (9.4–10.7 g/dL).

Compared with older adults, rates of LTFU were higher in the youngest age group in all seven countries, reaching statistical significance in unadjusted analyses in three countries (Côte d’Ivoire (p=0.005), Mozambique (p<0.001), and Tanzania (p=0.005)) (Table 3). Even after adjusting for baseline demographic and clinical characteristics, rates of LTFU were 1.66–2.45 times as high in the youngest compared with the oldest age group in these three countries (Côte d’Ivoire [p=0.001], Mozambique [p=0.002], and Tanzania [p<0.001]).

In two countries (Swaziland and Uganda), the oldest age group had significantly higher rates of documented mortality than younger age groups (Table 3), and older age remained a significant predictor of mortality even in multivariable analyses.

## Discussion

The three main findings based on the experience of the seven African countries are as follows: 1) adolescents and young adults differed significantly from older adults in ART enrollment characteristics; 2) adolescents and young adults tended to have higher LTFU rates; and 3) in two countries (Uganda and Swaziland), adults ≥50 years had higher documented mortality rates.

Adolescent and young adult ART enrollees were almost exclusively female, commonly pregnant, unmarried, and unemployed. The observation that median weight was lowest among adolescents and young adults could be explained by expected weight-for-age growth, sex differences in weight, or undernutrition. Similarly, the observation that median hemoglobin tended to be lowest in the youngest age group might reflect predominantly female sex or higher prevalence of undernutrition.

Available data suggest that this group of predominantly female adolescent and young adult ART enrollees represents a socially vulnerable population (2). Although rates of HIV-related mortality and HIV incidence have declined globally since 2005, mortality has increased and HIV incidence remained relatively stable among adolescents, with the majority of adolescent deaths and new HIV infections occurring in sub-Saharan Africa (2). In African countries with generalized epidemics, being young, female, and unemployed increases the risk for voluntary or coerced sexual contact with older, HIV-infected men (2); this might partly explain HIV infection at a young age among some of the female adolescent and young adult ART enrollees described in this report. Factors that possibly explain high LTFU rates among adolescent and young adult ART enrollees might include stigma (2), lack of money for transport (5), child care responsibilities, and migration for work (6). LTFU from ART is associated with significant increases in mortality risk (7). A recent meta-analysis suggests that 20%–60% of patients lost to follow-up die, with most of these deaths occurring after default from ART (7). Therefore, difficulties in preventing LTFU among adolescent and young adults on ART might be a contributor to HIV-related mortality in this age group. Suboptimal ART adherence among adolescents might also be contributing to adolescent mortality (1).

High rates of LTFU among adolescent and young adult ART enrollees is also concerning from a prevention perspective, because LTFU patients are at risk for transmitting HIV to seronegative partners once ART is discontinued and viral load no longer suppressed (8). High rates of LTFU among young women, among whom the prevalence of pregnancy is high, also increases the likelihood of mother-to-child HIV transmission.

Adult ART enrollees aged ≥50 years were mostly male, commonly married, and employed. In two countries, this age group had higher documented mortality, similar to findings in other studies (9). Higher mortality in this oldest age group should probably be expected because of higher background rates of mortality in the older general population. However, HIV-related reasons for higher mortality in the oldest age group might include slower ART-induced CD4 restoration among older patients (3) or incidence of HIV-associated noncommunicable diseases, especially atherosclerotic disease (10).

The findings in this report are subject to at least four limitations. First, missing data might have introduced nondifferential measurement error. Second, because of differences in cohort size, there was greater power to detect covariate effect sizes in Côte d’Ivoire, Nigeria, Swaziland, and Mozambique than in Zambia, Uganda, and Tanzania. Third, in Zambia, Uganda, and Tanzania, clinics were purposefully selected, limiting generalizability of findings. Finally, limited active tracing for defaulting patients might have resulted in overestimates of LTFU and underestimates of mortality.

The main finding of this report is that adolescent and young adult ART enrollees differ significantly from older adults in demographic and clinical characteristics and are at higher risk for LTFU. Effective interventions to reduce LTFU for adolescent and young adult ART enrollees could help reduce mortality and HIV incidence in this age group.

## Figures and Tables

**TABLE 1:** Summary of sampling strategies to select cohorts of enrollees for antiretroviral therapy (ART) — seven African countries, 2004–2013

Region and country	Stage 1: Selection of study facilities							Stage 2: Selection of study patients					
	
	No. of ART clinics	No. of ART enrollees at ART clinics	Clinic eligibility criteria for study	No. of study-eligible clinics	Estimated no. of study-eligible adult ART enrollees at study-eligible clinics	Site sampling technique	No. of clinics selected	Age-eligibility criteria (age at ART initiation)	ART enrollment years	Patient sampling technique at selected study clinics	Planned sample size	No. of eligible patient charts abstracted	Date of data collection
**West Africa**													
Côte d’Ivoire	124 by Dec 2007	36,943	Enrolled ≥50 adults by Dec 2007	78	36,110	PPS	34	Adults aged ≥15 yrs	2004–2007	SRS	4,000	3,682	Nov 2009–March 2010
Nigeria	178 by Dec 2009	168,335	Enrolled ≥50 adults by Dec 2009	139	167,438	PPS	35	Adults aged ≥15 yrs	2004–2012	SRS	3,500	3,496	Dec 2012–Aug 2013
**Southern Africa**													
Swaziland	31 by Dec 2009	50,767	All ART initiation sites eligible	31	50,767	PPS	16	Adults aged ≥15 yrs	2004–2010	SRS	2,500	2,510	Nov 2011– Feb 2012
Mozambique	152 by Dec 2006	43,295	Enrolled ≥50 adults by Dec 2006	94	42,234	PPS	30	Adults aged ≥15 yrs	2004–2007	SRS	2,600	2,596	Sept–Nov 2008
Zambia	322 by Dec 2007	65,383	Enrolled ≥300 adults by Dec 2007	129*	58,845*	Purposeful	6	Adults aged ≥15 yrs	2004–2009	SRS	1,500	1,214†	April–July 2010
**East Africa**													
Uganda	286 by Dec 2007	45,946	Enrolled ≥300 adults by Dec 2007	114*	41,351*	Purposeful	6	Adults aged ≥15 yrs	2004–2009	SRS	1,500	1,466§	April–July 2010
Tanzania	210 by Dec 2007	41,920	Enrolled ≥300 adults by Dec 2007	85	37,728*	Purposeful	6	Adults aged ≥18 yrs	2004–2009	SRS	1,500	1,457¶	April–July 2010
**Total**		**452,589**		**670**	**434,473**		**133**				**17,100**	**16,421**	

**Abbreviations:** PPS = probability-proportional-to-size; SRS = simple random sampling.

* Estimates based on available published data.

† In Zambia, from 1,457 records sampled, 243 were excluded because of noncompliance with simple random sampling procedures at one site.

§ In Uganda, from 1,472 records samples, six patients were excluded because of absence of age data at ART initiation.

¶ In Tanzania, from 1,458 records samples, one patient was excluded because of absence of age data at ART initiation.

**TABLE 2:** Demographic and clinical characteristics of patients at initiation of antiretroviral therapy (ART) — seven African countries, 2004–2012*

Characteristic and age group (yrs)	Côte d’Ivoire† (N = 3,682)		Nigeria† (N = 3,496)		Swaziland† (N = 2,510)		Mozambique† (N = 2,596)		Zambia (N = 1,214)		Tanzania (N = 1,457 )		Uganda (N = 1,466)	
**Age at ART initiation (No. and %)**														
15–24	188	5%	399	11%	398	16%	284	12%	95	8%	83	6%	95	6%
25–49	3,087	83%	2,805	81%	1,759	70%	2,069	79%	1,000	82%	1,198	82%	1,261	86%
≥50	407	12%	292	9%	353	14%	243	10%	119	10%	176	12%	110	8%
**Female (No. and %)**														
15–24	166	87%	366	92%	326	82%	45	86%	82	86%	73	88%	77	81%
25–49	2,077	68%	1,808	64%	1,120	64%	838	60%	599	60%	813	68%	837	66%
≥50	179	46%	146	51%	175	49%	137	48%	45	38%	87	49%	50	45%
p–value	**<0.001** §		**<0.001**		**<0.001**		**<0.001**		**<0.001**		**<0.001**		**<0.001**	
**Among females, pregnant (No. and %)**														
15–24	4	3%	56	16%	82	26%	61	30%	15	32%	—		25	18%
25–49	64	4%	188	10%	117	11%	138	14%	56	12%	—		102	9%
≥50	0	0%	0	0%	2	1%	0	0%	0	0%	—		0	0%
p-value	0.567		**<0.001**		**<0.001**		**0.002**		**0.003**				**<0.001**	
**Married/Civil union (No. and %)**														
15–24	41	27%	177	43%	85	28%	99	41%	38	46%	28	41%	21	34%
25–49	1,393	50%	1,795	64%	725	47%	999	55%	520	60%	505	53%	431	43%
≥50	202	54%	200	67%	190	65%	113	55%	67	64%	71	49%	40	43%
Missing	414	11%	86	2%	384	15%	233	9%	166	14%	299	21%	313	21%
p-value	**<0.001**		**<0.001**		**<0.001**		**0.001**		**0.022**		0.115		0.354	
**Employed (No. and %)**														
15–24	59	47%	91	30%	68	31%	28	14%	—		—		—	
25–49	1,394	63%	1,541	66%	551	48%	860	49%	—		—		—	
≥50	148	53%	165	70%	73	32%	104	56%	—		—		—	
Missing	1,081	29%	420	12%	925	37%	328	13%						
p-value	**<0.001**		**<0.001**		**<0.001**		**<0.001**							
**Baseline weight (No. and median [kg])**														
15–24	162	49.0	371	52.0	356	58.0	223	50.0	83	49.0	80	48.2	86	52.7
25–49	2,743	53.0	2589	57.0	1575	60.0	1,658	54.5	882	53.0	1,163	51.1	1,145	55.0
≥50	351	54.0	274	57.0	301	59.9	180	52.5	108	55.0	172	50.2	101	56.0
Missing	426	12%	262	7%	278	11%	535	21%	141	12%	42	3%	134	9%
p-value	**0.005**		**<0.001**		**0.024**		**0.015**		**0.001**		0.296		**0.001**	
**WHO clinical stage 4 (No. and %)**														
15–24	25	18%	25	5%	22	6%	32	20%	11	13%	20	29%	12	14%
25–49	462	22%	197	8%	218	13%	205	15%	96	11%	257	27%	137	12%
≥50	67	25%	24	11%	53	16%	22	15%	5	5%	48	35%	11	12%
Missing	1,101	30%	232	7%	290	12%	979	38%	157	13%	293	20%	164	11%
p-value	0.468		**0.012**		**<0.001**		0.066		0.100		**<0.001**		0.551	
**Baseline CD4 count (No. and median [cells/** *μ* **L])**														
15–24	165	122	320	192	359	158	249	175	69	147	50	175	76	161
25–49	2,811	136	2321	157	1618	141	1,794	157	701	128	933	126	1,011	133
≥50	367	132	244	142	319	160	211	133	79	158	137	160	79	147
Missing	339	9%	611	17%	214	9%	342	13%	365	30%	337	23%	300	20%
p-value	0.216		**0.004**		0.139		0.077		0.704		0.243		0.501	
**Baseline hemoglobin (No. and median [g/dL])**														
15–24	156	10.0	190	10.3	229	10.7	211	9.4	52	10.1	37	9.6	55	11.5
25–49	2,646	9.9	1,365	10.3	1165	11.2	1,515	10.2	582	10.6	648	10.2	748	11.9
≥50	347	9.9	145	10.8	218	11.6	173	10.6	70	11.6	90	10.9	62	12.1
Missing	533	14%	1,796	51%	898	36%	697	27%	510	42%	682	47%	601	41%
p-value	0.524		0.690		**<0.001**		**<0.001**		**0.002**		**0.028**		0.306	

**Abbreviation:** WHO = World Health Organization.

* Although the study captured patient follow-up time through 2013, all patients started ART during the period 2004–2012.

† Proportions from Côte d’Ivoire, Nigeria, Swaziland, and Mozambique are weighted to account for sampling design.

§ Bold-typed p-values are statistically significant (p<0.05).

**TABLE 3:** Association between age group at initiation of antiretroviral therapy and rates of loss to follow-up and death — seven African countries, 2004–2013

Country	Age group (yrs)	No.	Lost to follow-up							Died						
	
			Rate (per 100)	Crude			Adjusted			Rate (per 100)	Crude			Adjusted		
			
				HR	(95% CI)	p-value	AHR*	(95% CI)	p-value		HR	(95% CI)	p-value	AHR*	(95% CI)	p-value
**Côte d’Ivoire**																
	≥50	407	14.5	1.00	—	—	1.00	—	—	4.2	1.00	—	—	1.00	—	—
	25–49	3,087	17.5	1.21	(0.92–1.59)	0.171	1.33	(1.00–1.77)	**0.052** †	2.9	0.68	(0.45–1.05)	**0.077**	0.76	(0.51–1.12)	0.155
	15–24	188	23.0	1.54	(1.15–2.04)	**0.005**	1.66	(1.24–2.22)	**0.001**	3.8	0.87	(0.37–2.03)	0.732	0.97	(0.43–2.18)	0.935
**Nigeria**																
	≥50	399	15.3	1.00	—	—	1.00	—	—	1.5	1.00	—	—	1.00	—	—
	25–49	2,805	13.7	0.91	(0.70–1.18)	0.446	0.94	(0.73–1.22)	0.640	1.1	0.79	(0.43–1.46)	0.441	0.89	(0.47–1.68)	0.714
	15–24	292	16.5	1.09	(0.79–1.50)	0.604	1.04	(0.75–1.44)	0.818	0.8	0.51	(0.20–1.34)	0.166	0.74	(0.30–1.86)	0.514
**Swaziland** §																
	≥50	353	11.0	1.00	—	—	1.00	—	—	3.0	1.00	—	—	1.00	—	—
	25–49	1,759	11.4	1.06	(0.91–1.23)	0.452	0.99	(0.81–1.20)	0.887	1.9	0.66	(0.46–0.93)	**0.021**	**0.56**	(0.39–0.81)	**0.006**
	15–24	398	13.2	1.26	(0.94–1.70)	0.113	1.22	(0.89–1.68)	0.198	1.9	0.65	(0.46–0.92)	**0.018**	**0.58**	(0.38–0.90)	**0.019**
**Mozambique**																
	≥50	243	16.4	1.00	—	—	1.00	—	—	3.8	1.00	—	—	1.00	—	—
	25–49	2,069	14.4	0.96	(0.78–1.18)	0.686	1.02	(0.79–1.32)	0.872	3.2	0.94	(0.55–1.59)	0.805	1.10	(0.62–1.96)	0.733
	15–24	284	28.4	1.80	(1.46–2.21)	**<0.001**	1.76	(1.27–2.43)	**0.002**	5.0	1.40	(0.72–2.71)	0.296	1.33	(0.72–2.45)	0.339
**Zambia**																
	≥50	95	21.4	1.00	—	—	1.00	—	—	3.6	1.00	—	—	1.00	—	—
	25–49	1,000	21.7	1.01	(0.75–1.37)	0.928	0.94	(0.69–1.29)	0.722	2.3	0.63	(0.29–1.33)	0.223	0.66	(0.30–1.47)	0.312
	15–24	119	25.6	1.14	(0.75–1.74)	0.539	1.21	(0.78–1.89)	0.393	5.1	1.32	(0.49–3.51)	0.582	1.26	(0.43–3.71)	0.679
**Tanzania**																
	≥50	83	13.0	1.00	—	—	1.00	—	—	8.0	1.00	—	—	1.00	—	—
	25–49	1,198	17.8	1.36	(0.98–1.90)	**0.067**	1.47	(1.05–2.06)	**0.024**	6.4	0.80	(0.52–1.23)	0.309	0.90	(0.58–1.42)	0.661
	15–24	176	30.1	2.01	(1.24–3.25)	**0.005**	2.45	(1.50–4.01)	**<0.001**	13.5	1.37	(0.70–2.70)	0.358	1.40	(0.69–2.82)	0.354
**Uganda**																
	≥50	95	6.0	1.00	—	—	1.00	—	—	2.8	1.00	—	—	1.00	—	—
	25–49	1,261	7.6	1.29	(0.76–2.17)	0.346	1.37	(0.81–2.34)	0.240	1.0	0.35	(0.15–0.80)	**0.013**	**0.31**	(0.13–0.76)	**0.010**
	15–24	110	7.1	1.18	(0.57–2.44)	0.664	1.19	(0.56–2.51)	0.647	1.0	0.34	(0.07–1.66)	0.184	**0.25**	(0.05–1.29)	**0.098**

**Abbreviations:** HR = hazard ratio; CI = confidence interval; AHR = adjusted hazard ratio.

* All variables presented in the table were included in the multivariable model for each country.

† Bold-typed p-values are statistically significant (p<0.05) or borderline significant (p=0.05–0.10).

§ In Swaziland, the study was designed to assess the effect of interfacility transfer of stable patients (down-referral) on risk for loss to follow-up, and this time-varying covariate was included in the multivariable model in addition to variables presented in the table.

## Footnotes

* Information available at http://www.unaids.org/en/media/unaids/contentassets/documents/epidemiology/2013/gr2013/UNAIDS_Global_Report_2013_en.pdf.

† Sources: Kasedde S, Luo C, McClure C, Chandan U. Reducing HIV and AIDS in adolescents: opportunities and challenges. Curr HIV/AIDS Rep 2013;10:159–68; and UNAIDS. Report on the Global AIDS Epidemic, 2012, unpublished estimates; Spectrum 2012.

§ Information available at http://www.unaids.org/sites/default/files/en/media/unaids/contentassets/documents/epidemiology/2012/gr2012/JC2434_WorldAIDSday_results_en.pdf.
